# Functional Parameters of Prestin Are Not Correlated With the Best Hearing Frequency

**DOI:** 10.3389/fcell.2021.638530

**Published:** 2021-05-11

**Authors:** Zhongying Wang, Qingping Ma, Jiawen Lu, Xiaochen Cui, Haifeng Chen, Hao Wu, Zhiwu Huang

**Affiliations:** ^1^Department of Otolaryngology-Head and Neck Surgery, Shanghai Ninth People’s Hospital, Shanghai Jiao Tong University School of Medicine, Shanghai, China; ^2^Ear Institute, Shanghai Jiao Tong University School of Medicine, Shanghai, China; ^3^Shanghai Key Laboratory of Translational Medicine on Ear and Nose Diseases, Shanghai, China; ^4^State Key Laboratory of Microbial Metabolism, Joint International Research Laboratory of Metabolic and Developmental Sciences, Department of Bioinformatics and Biostatistics, National Experimental Teaching Center for Life Sciences and Biotechnology, School of Life Sciences and Biotechnology, Shanghai Jiao Tong University, Shanghai, China; ^5^Shanghai Center for Bioinformation Technology, Shanghai, China

**Keywords:** prestin orthologs, high-frequency hearing limit (*F*_max_), non-linear capacitance, whole cell patch-clamp, 3D protein structure

## Abstract

Among the vertebrate lineages with different hearing frequency ranges, humans lie between the low-frequency hearing (1 kHz) of fish and amphibians and the high-frequency hearing (100 kHz) of bats and dolphins. Little is known about the mechanism underlying such a striking difference in the limits of hearing frequency. Prestin, responsible for cochlear amplification and frequency selectivity in mammals, seems to be the only candidate to date. Mammalian prestin is densely expressed in the lateral plasma membrane of the outer hair cells (OHCs) and functions as a voltage-dependent motor protein. To explore the molecular basis for the contribution of prestin in hearing frequency detection, we collected audiogram data from humans, dogs, gerbils, bats, and dolphins because their average hearing frequency rises in steps. We generated stable cell lines transfected with human, dog, gerbil, bat, and dolphin prestins (hPres, dPres, gPres, bPres, and nPres, respectively). The non-linear capacitance (NLC) of different prestins was measured using a whole-cell patch clamp. We found that the *Q*_max_/*C*_lin_ of bPres and nPres was significantly higher than that of humans. The *V*_1__/__2_ of hPres was more hyperpolarized than that of nPres. The *z* values of hPres and bPres were higher than that of nPres. We further analyzed the relationship between the high-frequency hearing limit (*F*_max_) and the functional parameters (*V*_1__/__2_, *z*, and *Q*_max_/*C*_lin_) of NLC among five prestins. Interestingly, no significant correlation was found between the functional parameters and *F*_max_. Additionally, a comparative study showed that the amino acid sequences and tertiary structures of five prestins were quite similar. There might be a common fundamental mechanism driving the function of prestins. These findings call for a reconsideration of the leading role of prestin in hearing frequency perception. Other intriguing kinetics underlying the hearing frequency response of auditory organs might exist.

## Introduction

Hearing frequency resolution is a result of selective pressure for accurate and instantaneous localization of the source of brief sounds. Behavioral studies have shown that the high-frequency hearing limit (*F*_max_) ranges enormously across classes (Masterton et al., 1969). Most fishes can only detect sounds below 0.74 kHz (Popper and Fay, 1997). In some species of frogs, *F*_max_ values are nearly five times higher than that of fish (Ji et al., 2018). *F*_max_ tends to increase phylogenetically from fish to amphibians and mammals. Humans have an *F*_max_ value of approximately 20 kHz, while bats and dolphins can hear sounds with frequencies near 150 kHz (Heffner et al., 2013; Nachtigall et al., 2016; Simmons et al., 2017). In fact, the hearing frequency of humans is lower than that of other mammals, like dogs and gerbils, whose *F*_max_ is 45 and 60 kHz, respectively (Heffner et al., 1994; Ogawa et al., 2018). Little is known about the mechanism underlying such a large hearing frequency difference among them.

Prestin, also named SLC26A5, belongs to the solute carrier 26 (SLC26) gene family. It shares the structure of the SLC26A protein family: approximately 14 transmembrane (TM) domains are linked by intra- and extracellular loops (Chang et al., 2019). This motor protein is essential for the electromotility of the outer hair cells (OHCs) and can be measured by its robust voltage-dependent non-linear capacitance (NLC; Brownell et al., 1985; Santos-Sacchi, 1991). Compared with other vertebrates whose OHCs do not possess electromotility, mammals have a greater NLC and a higher frequency hearing limit (Tan et al., 2011). When not expressed, mammals experience a 50-dB threshold shift, and the cochlea fails to function as a frequency analyzer (Cheatham et al., 2004). Mutations in this gene lead to a loss in cochlear function in humans (Liu et al., 2003). Many studies suggest that prestin plays a significant role in high-frequency detection (Bai et al., 2019; Morell et al., 2020).

**FIGURE 1:**
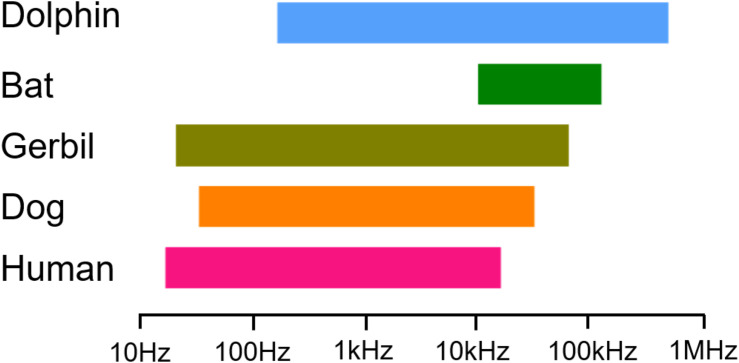
Data showing the low- and high-frequency sensitivity of humans, dogs, gerbils, bats, and dolphins through audiograms collected.

Herein, we collected data on the low- and high-frequency sensitivities of humans, dogs, gerbils, bats, and dolphins through audiograms obtained from previous studies (Au and Moore, 1990; Heffner et al., 1994; Simmons et al., 2017; Ogawa et al., 2018; Hunter et al., 2020). Our choice of these particular mammals as experimental subjects was based on their gradually increasing *F*_max_ values, which might be related to the function of their prestins (Figure 1). We analyzed the amino acid sequences of human prestin (hPres), dog prestin (dPres), gerbil prestin (gPres), bat prestin (bPres), and dolphin prestin (nPres) for the purpose of seeking the molecular basis for the contribution of prestin in hearing frequency detection. NLC is often used to evaluate the function of prestin not only due to its link to electromotility but also because it can be easily assayed experimentally (Santos-Sacchi, 1991). In order to investigate the function of prestin from different mammals and to put them in the same circumstance, we generated stable cell lines expressing hPres, dPres, gPres, bPres, and nPres. The NLC of five prestin orthologs was measured using patch-clamp technology. The functional parameters *Q*_max_/*C*_lin_, *z*, and *V*_1__/__2_ of NLC were analyzed in the present study. We also predicted the prestin tertiary structures of five species to obtain an overall view of the protein conformation among different species. Our goal was to investigate the relationship between prestin function and hearing frequency capability in mammals.

## Materials and Methods

### Cloning and Analyses of Prestin Orthologs

We obtained the prestin coding region of humans (*Homo sapiens*, 375611), dogs (*Canis lupus familiaris*, 483274), gerbils (*Meriones unguiculatus*, 110554811), vampire bats (*Desmodus rotundus*, 112301880), and bottlenose dolphins (*Tursiops truncates*, 101316391) using BLAST analyses on the Ensembl and NCBI genomic databases. The genomic sequence data of humans, dogs, gerbils, bats, and dolphins were utilized, and the resulting deduced full coding cDNAs were synthesized (HuaGene, China). The correct orientation and reading frames were verified by sequence analyses. Ortholog comparisons were performed using UniProt, CLUSTALW, and ESPript 3.0. All the constructs were verified by sequencing.

### Collection of *F*_max_ Data in Mammals

The *F*_max_ values defining the highest frequency audible at 60 dB were collected from five mammals (Figure 1). The first is the *F*_max_ of humans with a value of 20 kHz (Hunter et al., 2020) followed by dogs with an *F*_max_ value of 45 kHz (Heffner et al., 1994) and gerbils with an *F*_max_ value of 60 kHz (Ogawa et al., 2018). We also chose two high-frequency hearing mammals—vampire bats, which have an *F*_max_ value of 113 kHz (Heffner et al., 2013), and bottlenose dolphins, which have an *F*_max_ value of approximately 150 kHz (Au and Moore, 1990). Our choice of these five mammals is based entirely upon their cascading *F*_max_ values.

### Generation of Stable Cell Lines That Express hPres, dPres, gPres, bPres, and nPres

CRISPR/Cas9-mediated gene editing was used for AAVS1 site-specific integration. The entire coding region of humans, dogs, gerbils, bats, and dolphins was synthesized and cloned into the expression vector. The correct orientation and reading frames were verified by sequencing analysis. The vector was generated by assembling the PCR-amplified fragments by restriction digestion and ligation, and was designed for the expression of prestin that is linked to enhanced GFP (EGFP) using the CMV promoter. This step helped to verify the transfection efficiency and identify cells during electrophysiological recording. The HEK293T cells were cultured in Dulbecco’s modified Eagle’s medium (Invitrogen, Carlsbad, CA, United States) supplemented with 10% fetal bovine serum (Invitrogen). Then, they were co-transfected with sgRNA/Cas9 and donor plasmids. Puromycin was added to the culture medium 24 h after transfection for screening purposes. Fluorescence microscopy was used to examine the membrane targeting of prestin and EGFP. Cells transfected only with the EGFP vector were used as a negative control.

### Confocal Imaging

The cells from the stable cell line at passage 6 were cultured for 12 h before immunodetection. The cells were rinsed with phosphate-buffered saline once. They were then fixed and permeabilized with 4% paraformaldehyde and 1% Triton X-100 for 30 min. After that, the cells were washed twice for 15 min each. Confocal imaging was conducted with a laser scanning microscope (Leica Microsystems, Germany) using a 63X oil immersion objective. Fluorescence intensity was also measured and compared through five cell lines to ensure a similar protein expression level.

### Electrophysiology

The patch pipettes were pulled from thick-walled borosilicate glass (World Precision Instruments) using a Narishige puller (model PP-830) to resistances of 5–8 MΩ and coated with dental wax for hair cells. Whole-cell voltage-clamp recordings were performed with an EPC-10/2 (HEKA Electronics, Lambrecht/Pfalz, Germany) patch-clamp amplifier and Pulse software (HEKA). The cells were held at −80 mV. Offline analysis was performed mainly using the Igor Pro 5.0 software (Wavemetrics, Portland, OR, United States).

The HEK cells were detached with trypsin (Invitrogen) before the recordings. The detached cells were then bathed in an extracellular solution containing 120 mM NaCl, 20 mM TEA-Cl, 2 mM CoCl_2_, 2 mM MgCl_2_, 10 mM HEPES, and 5 mM glucose at pH 7.2. The osmolarity was adjusted to 300 mosmol/L with glucose. Recording pipettes were pulled with resistances of 2.5–5.0 MΩ and filled with an internal solution containing 140 mM CsCl, 2 mM MgCl_2_, 10 mM EGTA, and 10 mM HEPES. The NLC measurements were done on cultured cells with a robust membrane-associated EGFP expression. After rupturing, we selected cells whose membrane resistance was over 300 MΩ and showed normal *C*m and *R*m values.

The sine + DC software lock-in function of Patchmaster (HEXA) was used to obtain the voltage-sensor displacement currents and capacitance. A voltage protocol was designed that included both ramp and sine stimulation (800 Hz with a 10-mV amplitude). Sine waves were superimposed onto ramps from –150 to 100 mV for a duration of 300 ms. The NLC was fitted with the derivative of a Boltzmann function as follows:

$$C_{m}=\frac{Q_{\max}\,\alpha }{\exp\left[\alpha \,\left(V_{m}-V_{\frac{1}{2}}\right)\right]\,{\left(1+\exp\left[-\alpha \,\left(V_{m}-V_{\frac{1}{2}}\right)\right]\right)}^{2}}+C_{\text{lin}}$$

where *Q*_max_ is the maximum charge transfer, *V*_1__/__2_ is the voltage at half-maximum charge transfer, *C*_lin_ is the residual linear membrane capacitance, and α is the slope factor describing the voltage dependence. Here, *k* is the Boltzmann’s constant, *T* is the absolute temperature, *z* is the valence of charge movement, and e is the electron charge.

### Predicting Prestin Protein Tertiary Structure and Structural Superposition

The tertiary structures of hPres, dPres, gPres, bPres, and nPres were predicted online by the profile–profile matching algorithms implemented in the Phyre web server. The quality of the predicted proteins was estimated by the *E* value, where an *E* value < 0.001 corresponded to an estimated precision of >95% (Kelley and Sternberg, 2009).

## Results

### Amino Acid Sequence Comparing Among hPres, dPres, gPres, bPres, and nPres

**FIGURE 2:**
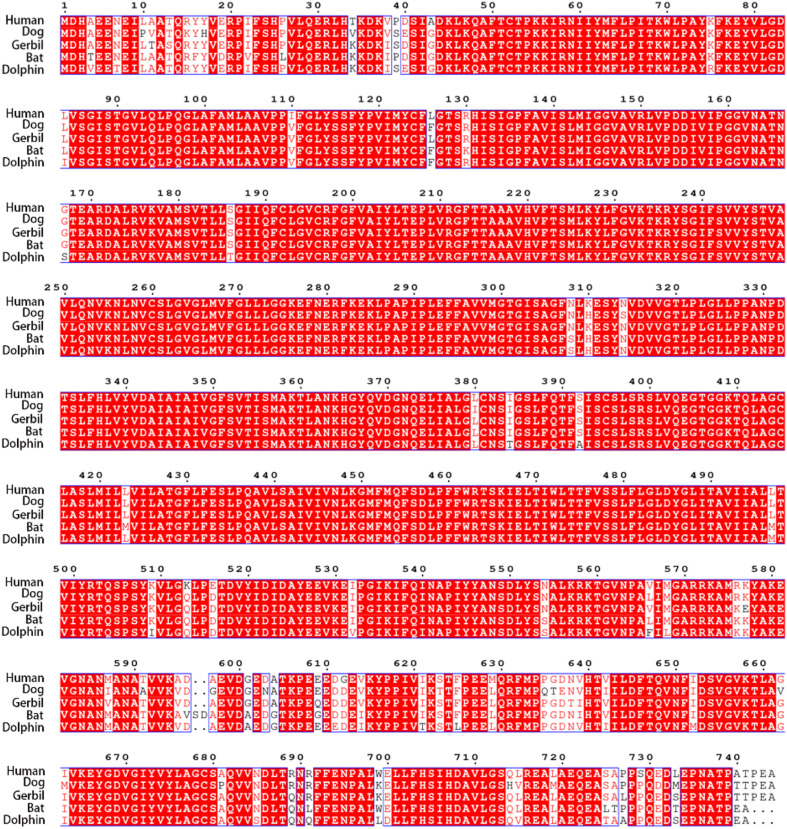
Alignment of the amino acid sequences of hPres, dPres, gPres, bPres, and nPres. Different colors have been used to represent the identity of each residue among the five mammals. Red block: full identity at a residue; red letter: partial identity at a residue; and black: complete disparity at a residue. Gaps in the aligned sequences are indicated by the dashed line.

Using the CLUSTALW method, the alignment of human, dog, gerbil, bat, and dolphin prestins is shown in Figure 2. This alignment revealed nearly 97% identity among these five species. Our alignment results were consistent with previous comparative peptide sequence analyses that stated that amino acid sequences of mammalian prestin are highly conserved. As shown in Table 1, hPres has 744 amino acids, dPres has 707 amino acids, gPres has 744 amino acids, bPres has 743 amino acids, and nPres has 741 amino acids. Comparisons of the deduced sequences from dPres, gPres, bPres, and nPres were made with hPres. From Table 1, the amino acids of hPres share 92% identity with dPres, 95% with gPres, 94% with bPres, and 93% with nPres. We noticed that the 7th, 384th, and 392nd amino acids in the prestin of humans, dogs, gerbils, and bats were *N*, *I*, and *S*. The same sites were *T*, *T*, and *A* in the prestin of dolphin. The 110th, 516th, and 629th sites of human prestin were *I*, *E*, and *M*, while the other four species all had *V*, *D*, and *L* in these three sites. The changes might cause the functional differences in their prestin.

**TABLE 1:** Amino acid sequence comparisons of human, dog, gerbil, bat, and dolphin prestin orthologs.

	**Human**	**Dog**	**Gerbil**	**Bat**	**Dolphin**
**Human** (744 residues)		92% (684)	95% (705)	94% (700)	93% (692)
**Dog** (707 residues)	92% (684)		90% (671)	90% (668)	89% (663)
**Gerbil** (744 residues)	95% (705)	90% (671)		92% (684)	92% (686)
**Bat** (743 residues)	94% (700)	90% (668)	92% (684)		94% (698)
**Dolphin** (741 residues)	93% (692)	89% (663)	92% (686)	94% (698)	

### Electrophysiological Measurements of hPres, dPres, gPres, bPres, and nPres

**FIGURE 3:**
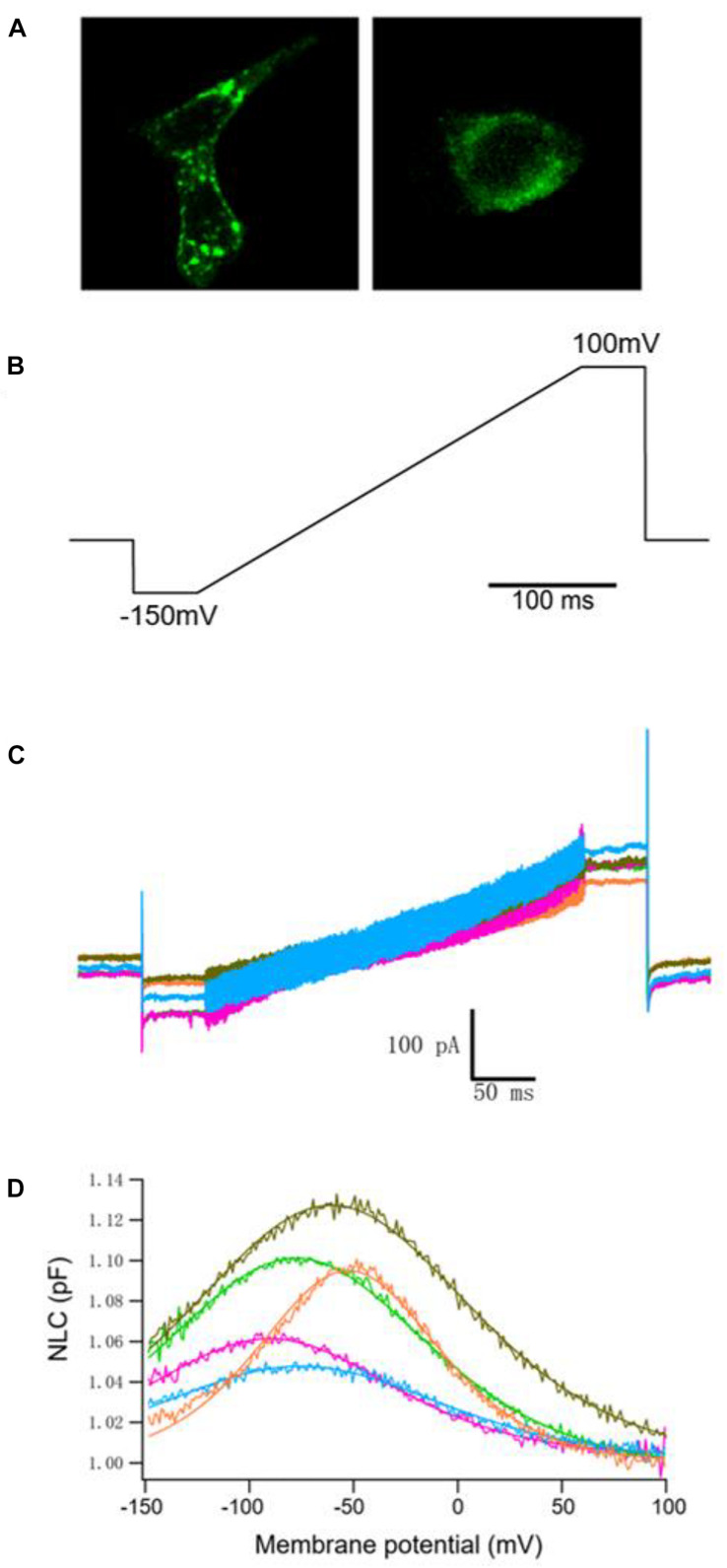
NLC obtained from hPres, dPres, gPres, bPres, and nPres transfected cells. **(A)** Confocal images showing the prestin-GFP expression. Scale bar: 10 μm. **(B)** Voltage ramps (300 ms in duration) varied from −150 to 100 mV. **(C)** Whole-cell currents of hPres-, dPres-, gPres-, bPres-, and nPres-transfected cells. Cells were held at −80 mV for current recordings. Red-hPres, orange-dPres, olive green-gPres, green-bPres, and blue-nPres. **(D)** NLC obtained from hPres-, dPres-, gPres-, bPres-, and nPres-transfected cells. Red—hPres, orange—dPres, olive green—gPres, green—bPres; and blue—nPres.

We examined the electrophysiological properties of hPres, dPres, gPres, bPres, and nPres from transfected cells. Considering transient transfections that might interfere with genes or disturb their transcription, we generated five stable cell lines to eliminate the negative influences (Li et al., 2019). All the five prestin clones exhibited cell surface expression, which was examined using confocal microscopy (Figure 3A). No difference was found in their expression levels according to the similar fluorescence intensity (Supplementary Figure 1).

The voltage stimulus we used for capacitance recordings consisted of a sine wave superimposed onto a voltage ramp from −150 to 100 mV, as shown in Figure 3B. From Figure 3C, we can see the currents of these five prestin-transfected cells. The NLC of the transfected cells was tested. As shown in Figure 3D, hPres, dPres, gPres, bPres, and nPres transfected cells obtained bell-shaped curves under the ramp voltage stimulus.

Using the first derivative of the Boltzmann function, four parameters (*Q*_max_, *C*_lin_, *V*_1__/__2_, and *z*) of the NLC were obtained. Because HEK293T cells varied in cell size, which was correlated with the *C*_lin_ value, we normalized the *Q*_max_ to the *C*_lin_ before analysis.

**FIGURE 4:**
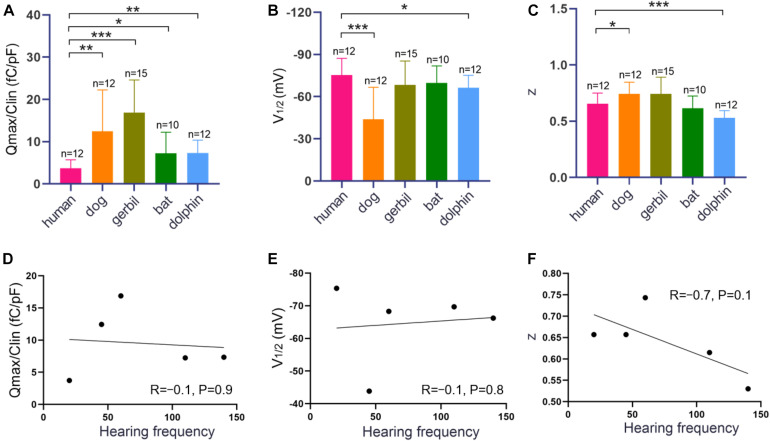
NLC functions of hPres, dPres, gPres, bPres, and nPres transfected cells. **(A–C)** The three parameters (*Q*_max_/*C*_lin_, *V*_1__/__2_, and *z*) derived from curve fittings with Boltzmann’s function for hPres (*n* = 12), dPres (*n* = 12), gPres (*n* = 15), bPres (*n* = 10), and nPres (*n* = 12). Data are expressed as mean ± S.D. **(D–F)** The correlation between three parameters (*Q*_max_/*C*_lin_, V_1__/__2_, and *z*) and F_max_ from the five mammals (* < 0.05, ** < 0.01, and *** < 0.001).

Measurements were collected from 12 hPres, 12 dPres, 15 gPres, 10 bPres, and 12 nPres transfected cells (Figures 4A–C). The means and SEMs of the hPres yielded values of *Q*_max_ = 38.8 ± 6.4 fC, *Q*_max_/*C*_lin_ = 3.7 ± 0.6 fC/pF, *V*_1__/__2_ = −75.4 ± 3.4 mV, and *z* = 0.66 ± 0.03. The means and SEMs of the dPres yielded values of *Q*_max_ = 142.3 ± 32.7 fC, *Q*_max_/*C*_lin_ = 12.4 ± 2.8 fC/pF, *V*_1__/__2_ = −43.8 ± 6.6 mV, and *z* = 0.74 ± 0.03. The means and SEMs of the gPres yielded values of *Q*_max_ = 279.4 ± 41.7 fC, *Q*_max_/*C*_lin_ = 16.9 ± 2 fC/pF, *V*_1__/__2_ = −68.3 ± 4.4 mV, and *z* = 0.74 ± 0.04. The means and SEMs of the bPres yielded values of *Q*_max_ = 97 ± 20 fC, *Q*_max_/*C*_lin_ = 7.2 ± 1.6 fC/pF, *V*_1__/__2_ = −69.7 ± 3.9 mV, and *z* = 0.62 ± 0.04. The means and SEMs of the nPres yielded values of *Q*_max_ = 63.8 ± 8.4 fC, *Q*_max_/*C*_lin_ = 7.3 ± 0.9 fC/pF, *V*_1__/__2_ = −66.2 ± 2.6 mV, and *z* = 0.53 ± 0.02. As shown in Figure 3D, the NLC magnitude of the gerbil prestin was the greatest. In comparison with the dog, gerbil, bat, and dolphin prestin, the *Q*_max_/*C*_lin_ of the human prestin was significantly lower (Figure 4A). The peak voltage of NLC (*V*_1__/__2_) of the human prestin significantly shifted toward the hyperpolarizing direction than the dog and dolphin prestins (Figure 4B). The *z* value of the human prestin was significantly lower than that of the dog prestin and higher than that of the dolphin prestin (Figure 4A).

To further explore the relationship between the functional parameters of NLC and high-frequency hearing, we collected the *F*_max_ values of five mammals through the audiograms from previous studies. Pearson’s correlation coefficients were then calculated between the *F*_max_ values and the functional parameters *Q*_max_/*C*_lin_, *z*, and *V*_1__/__2_. Figures 4D–F demonstrate that the values of *Q*_max_/*C*_lin_, *z*, and *V*_1__/__2_ have no significant correlation with the *F*_max_ values. All the data are shown in Table 2. All raw data are included in the Supplementary Material.

**TABLE 2:** All the NLC measurements in our study (mean ± SEM).

	***C*_lin_ (pF)**	***Q*_max_ (fC)**	***V*_1/2_ (mV)**	***z***	***Q*_max_/*C*_lin_ (fC/pF)**
Human (*n* = 12)	10.5 ± 0.7	38.8 ± 6.4	−75.4 ± 3.4	0.66 ± 0.03	3.7 ± 0.6
Dog (*n* = 12)	11.8 ± 1	142.3 ± 32.7	−43.8 ± 6.6	0.74 ± 0.03	12.4 ± 2.8
Gerbil (*n* = 15)	15.9 ± 1	279.4 ± 41.7	−68.3 ± 4.4	0.74 ± 0.04	16.9 ± 2
Bat (*n* = 10)	13.3 ± 0.7	97 ± 20	−69.7 ± 3.9	0.62 ± 0.04	7.2 ± 1.6
Dolphin (*n* = 12)	9.5 ± 1.5	63.8 ± 8.4	−66.2 ± 2.6	0.53 ± 0.02	7.3 ± 0.9

### Tertiary Structures of hPres, dPres, gPres, bPres, and nPres

**FIGURE 5:**
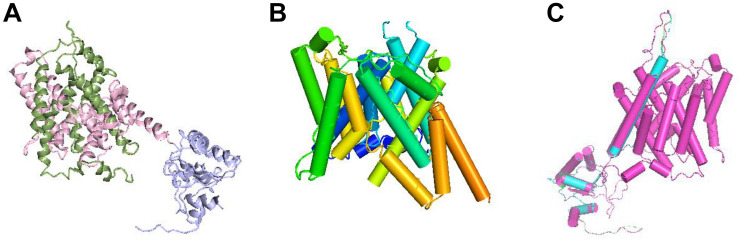
Structural superposition of the prestins. **(A)** Ribbon representation of the prestin protein; the *N*- and *C*-terminal halves of the TM domain are green and pink, respectively. The antisigma factor antagonist (STAS) domain is purple. **(B)** Transmembrane (TM) domain with α-helices shown as cylinders and labeled with the color spectrum. **(C)** Structural superposition of hPres, dPres, gPres, bPres, and nPres overlapping with each other.

The protein structure of prestin is depicted as ribbons in Figure 5A. From Figure 5B, the TM domain of prestin consisting of 14 α-helices of variable length is seen, including several short helices that do not span the entire width of the lipid bilayer. The human prestin was overlapped with the other four mammals’ prestins (Figure 5C).

## Discussion

Prestin is responsible for cochlear amplification and frequency selectivity in mammals, which underlies the mechanism of OHC electromotility (Dallos and Fakler, 2002; Liberman et al., 2002). This mechanism provides a gain of over 40 dB in hearing. It has also been characterized as being ultrafast and exhibiting no attenuation beyond 70 kHz (Frank et al., 1999). Such mechanical activities might contribute to cochlear amplification of some mammalian species with hearing capability well beyond the detectable frequency range of humans (Frank et al., 1999).

The hearing frequency of bats and dolphins is significantly higher than that of humans. The mechanism underlying this huge hearing frequency difference is still unknown. Whether prestin plays a leading role in this mechanism also remains a question. Voltage-dependent NLC is one of the unique features of prestin that is often used to measure the prestin function (Santos-Sacchi, 1991). NLC shows a bell-shaped curve that can be fitted with the first derivative of a two-state Boltzmann function (Santos-Sacchi, 1991; Oliver et al., 2001). We measured the NLC of high-frequency hearing mammals, bats, and dolphins. In our results, we noticed that the charge density (*Q*_max_/*C*_lin_) of the bat and dolphin prestins was significantly higher than that of the human prestin (*P* < 0.05, *P* < 0.01, and Figure 4A). Charge displacement manifests as a transient current at the onset and cessation of membrane potential steps. The total charge displaced at a given potential is the product of the total displaceable charge (*Q*_max_) in one of its two principal states. Previous studies indicate that the charge density of prestin coincides with the development of electromotility in OHCs (Seymour et al., 2016). Since we used the same cell line and the same way of protein expression, we believe that the level of the human, bat, and dolphin prestins on the cell surface was very close. We presume that the bat and dolphin prestins functioned more actively than the human prestin. Since the 7th, 384th, and 392nd amino acids in the prestin of dolphins were different from the other four, this might contribute to its optimal function in high-frequency detection.

Interestingly, when we explored the relationship between *Q*_max_/*C*_lin_ and the *F*_max_ values in five mammals, we found that there were no significant correlations between them (Figure 4D). Santos-Sacchi et al. used a wide-band macro-patch voltage clamp to drive prestin in mice and guinea pigs and revealed that its voltage-sensor charge movement was low pass in nature, being incapable of following high-frequency voltage changes. The frequency response mismatched its expected influence on cochlear amplification at very high frequencies (Santos-Sacchi and Tan, 2018). Their data show that when the frequency increases to 12.5 kHz, the electromotility fails to catch up. This result questions the long-held cycle-by-cycle hypothesis of cochlear amplification at very high frequencies. We speculated that the involvement of prestin might not be the only mechanism underlying high-frequency detection. Below the hearing range of 12.5 kHz, prestin might function in a similar way between humans and high-frequency hearing mammals. Optical coherence tomography has been widely used to visualize movements within the intact organ of Corti. The low-frequency mechanical activities of supporting cells and motions at the Deiters cell interface deep within the cochlea are also changing our original view of the influence of prestin on mechanisms underlying cochlear amplification (Gao et al., 2014; Cooper and van der Heijdenb, 2018).

Previous studies have analyzed the relationships among hearing frequencies and the *z* values and *V*_1__/__2_ of prestin NLC. They found that the z values were positively associated with the best hearing frequencies, while the *V*_1__/__2_ values were negatively associated (Liu et al., 2014). In our study, we also found that the *V*_1__/__2_ human prestin was more hyperpolarized than the dolphin prestin (Figure 4B). However, we did not observe a significant correlation between the *V*_1__/__2_ values and the values of *F*_max_. According to the former theory, the depolarizing shift of *V*_1__/__2_ in high-frequency hearing mammals can affect the anion-binding capability of prestin, with a change in the kinetics of prestin activation, resulting in high-frequency hearing (Ashmore, 2008). However, in non-mammalian animals and non-placental mammals possessing lower best-hearing frequencies, the peak of their prestin NLC did not significantly shift in the direction of the negative potential (Tang et al., 2013).

Our analysis of the unitary charge valence showed a statistically significant higher *z* value of the human prestin than the dolphin prestin and a higher *z* value of the bat prestin than the dolphin prestin. The *z* value of the bat and human prestins did not show a significant difference. It seemed that dolphins possessed the highest *F*_max_ among the mammals and had the lowest *z* value. Although our data showed that the *z* values were negatively associated with the best hearing frequencies (*R* = −0.7), no statistical significance (*P* = 0.1) was found. A possible explanation for these measurement disparities may be the experimental differences among studies.

In addition to the electrophysiological results, we also predicted the 3D structures of prestin from different mammals to evaluate whether conformational changes are involved in their functional activities. Since no high-resolution structures have been reported for prestin yet, the molecular mechanism of this voltage-driven motor protein remains unknown. SLC26 family proteins are composed of a TM domain and a carboxy-terminal cytoplasmic STAS (sulfate transporter and anti-sigma factor antagonist) domain. The STAS domain is responsible for substrate transport (Shibagaki and Grossman, 2006) and protein–protein interplays (Lolli et al., 2016). All the five prestins fit the same model with high predicted accuracy and significant *E* values. The tertiary structures of these five prestin proteins were quite similar, as shown in Figure 5C. In Table 1, a comparison of the deduced sequences from dPres, gPres, bPres, and nPres was made with hPres. The hPres, dPres, gPres, bPres, and nPres share a high degree of similarity in their amino acid sequences and tertiary protein structures. However, the human prestin had totally different amino acids compared to the other four species in the 110th, 516th, and 629th sites, pointing that these changes were underlying molecular mechanisms of their frequency detection gap. In summary, our study provides new insights into the relationship between prestin and high-frequency hearing capability in mammals. We analyzed the prestin functions of humans, dogs, gerbils, bats, and dolphins to investigate their effect in hearing frequency detection. The prestin of mammals with higher hearing frequency functioned more actively than the human prestin. When we further explored the relationship between the NLC parameters and the *F*_max_ of the five species, no significant correlations were found. We speculate that prestin might not be the only mechanism underlying the hearing frequency detection. Other intriguing kinetics underlying the hearing frequency response of auditory organs might exist.

## Data Availability Statement

The original contributions presented in the study are included in the article/Supplementary Material, further inquiries can be directed to the corresponding author/s.

## Author Contributions

ZW and QM performed the experiments. JL, XC, and HC had been involved in analysis and interpretation of data. ZW and ZH worked on drafting the manuscript. ZH and HW designed the experiments and revised the manuscript critically. All authors agreed that all the questions related to the accuracy or integrity of the manuscript have been appropriately investigated and resolved, giving final approval of the version to be published.

## Conflict of Interest

The authors declare that the research was conducted in the absence of any commercial or financial relationships that could be construed as a potential conflict of interest.
