# Supplementary material for: Functional Parameters of Prestin Are Not Correlated With the Best Hearing Frequency
Source: Front Cell Dev Biol. 2021 May 11;9:638530. doi: 10.3389/fcell.2021.638530 (PMC8144510; doi:10.3389/fcell.2021.638530)
Supplement: Supplementary file 2 [file Data_Sheet_1.docx]

Supplementary fig. 1


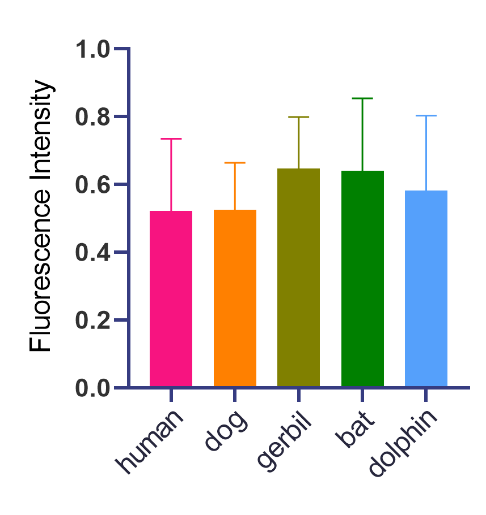
Fluorescence intensity measurements of five cell lines expressing hPres-eGFP, dPres-eGFP, gPres-eGFP, bPres-eGFP and nPres-eGFP. No significant difference was found in their fluorescence intensity. (P > 0.05) Data represents the mean ± SD from five cells per species.
